# Mixed Vaginitis in the Third Trimester of Pregnancy Is Associated With Adverse Pregnancy Outcomes: A Cross-Sectional Study

**DOI:** 10.3389/fcimb.2022.798738

**Published:** 2022-03-28

**Authors:** Huanrong Li, Mengting Dong, Wenjuan Xie, Wenhui Qi, Fei Teng, Huiyang Li, Ye Yan, Chen Wang, Cha Han, Fengxia Xue

**Affiliations:** ^1^ Department of Gynecology and Obstetrics, Tianjin Medical University General Hospital, Tianjin, China; ^2^ Tianjin Key Laboratory of Female Reproductive Health and Eugenic, Department of Gynecology and Obstetrics, Tianjin Medical University General Hospital, Tianjin, China

**Keywords:** mixed vaginitis, third trimester, pathogen, pregnancy outcomes, bacterial vaginosis

## Abstract

Mixed vaginitis is a complex vaginal dysbiosis that differs from single vaginitis. Vaginitis in the third trimester may lead to adverse maternal and neonatal outcomes. The clinical characteristics, microbiological characteristics, and adverse pregnancy outcomes of mixed vaginitis in late pregnancy are worth studying. Therefore, this study investigated the clinical and microbiological characteristics of vaginitis and adverse pregnancy outcomes of patients with mixed vaginitis. We studied 1,674 women in late pregnancy who attended the Tianjin Medical University General Hospital from November, 2019 to October, 2021. We administered standardized questionnaires, performed vaginal examination and sampling plus microscope examinations, and assessed follow-up pregnancy outcomes. We cultured the vaginal discharge of the patients with mixed vaginitis to isolate pathogens and performed antimicrobial susceptibility tests of the isolated pathogens. For the patients with peripartum infection, we collected a sample to isolate pathogens. Among the 1,674 women, 66 (3.9%) had mixed vaginitis. The independent risk factor for mixed vaginitis in late pregnancy was a history of vaginitis during early and middle pregnancy (OR = 5.637, 95% CI: 3.314–9.580). The signs of vaginal erythema (63.6% *vs.* 42.0%), yellow discharge (81.8% *vs*. 59.6%), and malodor (31.8% *vs*. 18.8%) (*P <*0.05) were significantly higher in patients with mixed vaginitis than in patients with single vaginitis. Bacterial isolates of the vaginal secretions of patients with mixed bacterial vaginitis were mainly the pathogens of aerobic vaginitis and bacterial vaginosis, such as *Gardnerella vaginalis*, *Streptococcus anginosus*, and *Staphylococcus epidermidis.* Pathogen isolation of the vaginal secretions of patients with mixed fungus and bacteria vaginitis mainly included *Candida albicans*, followed by *S. anginosus*, *Enterococcus faecalis*, *Staphylococcus hemolyticus*, *Staphylococcus aureus*, *Streptococcus agalactiae* and *Staphylococcus simulans*. Women with mixed vaginitis had an increased incidence and risk of peripartum infections (6.1% *vs*. 1.4%, *P* <0.05; OR = 3.985, 95% CI:1.214–13.079). *Escherichia coli* is the main pathogen that causes peripartum infection. Mixed vaginitis in late pregnancy is characterized by a severe and complex phenotype, complex vaginal dysbiosis, and a long course of vaginal dysbiosis. This can lead to an increased incidence and risk of peripartum infection. Therefore, more attention should be paid to patients with mixed vaginitis in the third trimester of pregnancy.

## Introduction

Vaginitis is the most common infectious disease of the female genital tract during the childbearing age. Common vaginal infections, namely, bacterial vaginosis (BV), aerobic vaginitis (AV), vulvovaginal candidiasis (VVC), and trichomoniasis, can lead to the adverse obstetrical and gynecological outcomes, such as cervicitis, pelvic inflammatory disease, postoperative infection, intrauterine infection, peripartum infection, and neonatal infection (Workowski et al., 2021).

Mixed vaginitis refers to two or more types of vaginitis occurring simultaneously (Sobel et al., 2013; Qi et al., 2021). Common mixed vaginitis includes BV + VVC, BV + AV, AV + VVC, trichomoniasis + AV, trichomoniasis + BV, trichomoniasis + VVC and VVC + AV + BV (Fan et al., 2013; Sobel et al., 2013; Narayankhedkar et al., 2015; Hillier et al., 2021). Existing studies have shown that mixed vaginitis is similar to single vaginitis and also leads to adverse gynecological and obstetrical outcomes (Govender et al., 1996; Wang et al., 2017; Abdul-Aziz et al., 2019). In addition, *in vitro* and *in vivo* studies have shown that in cases of mixed infection, the colonization and virulence of microorganisms and also the immune response of the host are enhanced compared with those observed in single infections, and the clinical phenotypes and adverse outcomes caused by microorganisms may be more severe in mixed infections than in single infections (Touyama et al., 1995; Peters and Noverr, 2013; Nash et al., 2016; Farrokhi et al., 2021). Therefore, research on the clinical characteristics of patients with mixed infections is very important.

Immune response changes during pregnancy. Studies have found that estrogen reduces the resistance of vaginal epithelial cells to pathogens (Fidel et al., 2000; Salinas-Muñoz et al., 2018) and progesterone enhances the adhesion of pathogens to the vaginal epithelium (Kalo and Segal, 1988). Another study found that estrogen and progesterone may enhance the virulence of pathogenic strains, leading to serious adverse outcomes (Robinson and Klein, 2012). Therefore, the colonization and virulence of pathogens in cases of mixed vaginitis and the immune response of the host during pregnancy may cause severe disease. Currently, there are few studies on mixed vaginitis during pregnancy, and there is still little understanding of the prevalence, disease phenotypes, and vaginal microflora characteristics of mixed vaginitis during pregnancy. Clarifying the disease characteristics of mixed vaginitis is important for understanding pathogenic mechanisms and for disease intervention. Single vaginitis, such as AV, BV, and VVC, during pregnancy is associated with an increase in the incidence of premature rupture of membranes (PROM), premature delivery, peripartum infection, and neonatal infection (Workowski and Bolan, 2015; Han et al., 2019). There is a need to examine whether mixed vaginitis increases the risk of adverse pregnancy outcomes. Therefore, this study assessed patients with mixed vaginitis in late pregnancy to summarize the clinical and microbiological characteristics of mixed vaginitis and its impact on pregnancy outcomes, which will provide suggestions for clinical diagnosis, treatment, and prophylaxis.

## Materials and Methods

### Participants

We conducted a cross-sectional study on pregnant women in their third trimester who attended the Tianjin Medical University General Hospital from November 2019 to October 2021. The inclusion criteria were as follows: 18–45 years old, intrauterine pregnancy, and intact fetal membrane. The exclusion criteria were as follows: unexplained vaginal bleeding, placenta previa, cervical cerclage, subjective induction of labor and abortion, antibiotic treatment within 4 weeks, sexual intercourse or use of vaginal medication within 3 days, mental or intellectual abnormalities, incomplete questionnaire information, and vulvar skin diseases. This project was approved by the ethics committee of Tianjin Medical University General Hospital (Ethical NO. IRB2020-WZ-194). All participants provided an informed consent before the study began.

### Questionnaire

The questionnaire was designed and administered by a professionally trained obstetrician based on our early research (Han et al., 2019). It recorded sociodemographic information, past medical history, symptoms and signs of genital tract infection, fertility history, personal hygiene habits, and sexual experience. A non-obstetrical clinician was invited to face validate the content of the questionnaire to make the research more rational. After face validation, another senior occupational obstetrician supervised the rationality of the questionnaire content prior to administration (Lynn, 1986). The pregnant women reviewed and answered the questionnaire in a location that was private and convenient to them. Each questionnaire was given a sequential number. After the pregnant women finished filling the questionnaire, the professionally trained obstetrician validated the content of the questionnaire in the part of sociodemographic information, past medical history, and fertility history with the maternal archive of pregnant women. Those parts is administered uniformly by web-based system of the government not only includes sociodemographic information, past medical history, and fertility history, but also medical records during pregnancy. Almost all feedback to the questionnaire were reliable. For the part of symptoms and signs of genital tract infection, personal hygiene habits, and sexual experience in questionnaire, the reliability was based on the feedback of the pregnant women. Gestational age was corrected based on the last menstrual period or ultrasound inspection of crown-rump length at 11–13  ^+6^ weeks (Committee Opinion No 700: Methods for Estimating the Due Date, 2017).

### Vaginal Examination, Sampling, and Laboratory Examination

The trained obstetrician used a nonlubricated sterile speculum and three long sterile cotton swabs to collect vaginal secretions from the lateral wall of the upper 1/3 of the vagina. The secretions were put into a sterile tube, marked with the same number as on the questionnaire. The tube was immediately transferred to the laboratory for pH testing, wet mount phase contrast microscopy (Olympus, Japan) (×400), and Gram-stained smear microscopy (×1,000) for the diagnosis of AV, BV, VVC, and trichomoniasis in the absence of any clinical information with the exception of the tube number. Blind laboratory examinations were performed by two experienced technicians. When the results from the two technicians were inconsistent, a third technician was consulted.

### Diagnosis Criteria

Lactobacillary grading (LBG) is based on the proportion of *Lactobacillus*. The following are the grades: LBG I: *Lactobacilli* are the dominant bacteria, with very few or no other bacteria; LBG IIa: mixed flora, but predominantly *lactobacilli*; LBG IIb: mixed flora, but the proportion of *lactobacilli* is significantly reduced due to the increased number of other bacteria; and LBG III: other bacteria are the dominant bacteria, with few or no *lactobacilli*. BV was diagnosed based on the Nugent score (Schwebke et al., 1996). Under the microscope (×1,000), the Nugent score was calculated by assessing three quantitative scores, namely, the presence of large Gram-positive bacilli (score of 0 to 4), small Gram-variable bacilli (score of 0 to 4), and curved Gram-variable bacilli (score of 0 to 2). A Nugent score of 7–10 indicated BV. An average of 10 high-power fields was used for the scoring. The diagnostic criteria for AV was based on the criteria of Donders et al. (2017). Five quantitative scores were given according to the proportion of lactobacilli (score 0 to 2), the number of white blood cells (score 0 to 2), the number of toxic white blood cells (score 0 to 2), the type of background bacteria (score 0 to 2), and the number of parabasal epithelial cells under the wet film through a phase-contrast microscope (×400) (score 0 to 2). An AV score ≥3 indicated AV. The diagnosis of VVC was based on the presence of hyphae and/or spores during wet preparation. Trichomoniasis was diagnosis based on the presence of active *Trichomonas vaginalis* on wet mount microscopy (Vieira-Baptista et al., 2021).

Mixed vaginal infection was defined as ≥2 kinds of vaginal infection simultaneously (Han et al., 2019). The diagnosis of AV + BV was based on Nugent score ≥7 and AV score ≥3 simultaneously. The diagnosis of VVC + BV was based on Nugent score ≥7 and presence of hyphae and/or spores during wet preparation simultaneously. The diagnosis of VVC + AV was based on AV score ≥3 and presence of hyphae and/or spores during wet preparation simultaneously. The diagnosis of trichomoniasis + AV was based on AV score ≥3 and presence of active *T. vaginalis* on wet mount microscopy simultaneously. The diagnosis of VVC + AV + BV was based on combined score of Nugent score ≥7, AV score ≥3, and the presence of hyphae and/or spores during wet preparation simultaneously. Vaginitis was treated according to international guidelines (Workowski and Bolan, 2015).

### Study Group Assignment

The women diagnosed with vaginitis were grouped according to the type of vaginitis (single vaginitis group: AV group, BV group, VVC group, trichomoniasis group; mixed vaginitis group: VVC + AV group, VVC + BV group, AV + BV group, AV + trichomoniasis group, and VVC + AV + BV group) and those without vaginitis were grouped into the nonvaginitis group. The mixed bacterial vaginitis group was the AV + BV group, and the mixed fungal and bacterial vaginitis group included the VVC + AV, VVC + BV, and VVC + AV + BV groups. Trichomoniasis mixed vaginitis group was the AV + trichomoniasis group.

### Bacterial and Fungal Isolation

Vaginal pathogens were isolated from patients with mixed vaginitis, and swabs were immediately sent to the laboratory. We performed bacterial isolation from vaginal discharge in patients with AV + BV. For the mixed fungal and bacterial vaginitis groups, namely, VVC + AV and VVC + BV groups, we performed bacterial and fungal isolation. The uterine cavity swabs, placental swabs, wound secretions swabs, and urine samples were cultured to isolate the pathogens.

For bacterial isolation, we used Columbia blood agar plates and MacConkey agar plates (bioMérieux, Marcy l’Etoile, France) overnight at 37°C in a constant CO_2_ temperature incubator (Thermo Fisher Scientific, USA). For fungal isolation, we used the Sabourand medium overnight at 37°C. A single colony was selected for identification and antimicrobial sensitivity testing.

### Blood Culture

Blood culture bottles (namely, anaerobic and aerobic culture vials) were incubated in a BD BACTEC™ FX automatic blood culture system at 37°C until culture positivity or a maximum of 5 days had passed. For the positive samples, we performed gram staining and culture on a suitable solid plate. Columbia blood agar plates (bioMérieux, Marcy l’Etoile, France) or MacConkey agar plates were incubated overnight at 37°C. A single colony was selected for identification and antimicrobial sensitivity testing.

### Strain Identification

We used matrix-assisted laser desorption/ionization time-of-flight mass spectrometry (MALDI-TOF-MS) to identify the species according to the manufacturer’s instructions.

### Antimicrobial Susceptibility Test

We used the disk diffusion method (K–B method) to test antimicrobial susceptibility on Mueller–Hinton agar plates. For gram-negative organisms, we used VITEK 2 GN 334 and VITEK 2 GN335 cards (bioMérieux, Marcy l’Etoile, France) on a VITEK 2 compact automated susceptibility testing system to perform the susceptibility tests. For gram-positive organisms, we used the VITEK 2 AST-GP69 card on a VITEK 2 compact instrument to perform the susceptibility tests. For the criteria for determining bacterial drug resistance, we referenced the M100-S26 criteria published by the American Clinical and Laboratory Standards Institute (CLSI) in 2016 to determine bacterial drug resistance. Further, we used ATB™ FUNGUS3 (bioMérieux, Marcy l’Etoile, France) to perform the susceptibility tests of fungi according to the manufacturer’s instructions.

### Quality Control

For the quality control, we used *E. coli* ATCC25922, *Klebsiella pneumoniae* ATCC700603 and *Pseudomonas aeruginosa* ATCC27853. All strains were purchased from the Clinical Laboratory Center of the National Health Commission of China.

### Pregnancy Outcomes

Maternal outcomes included preterm birth (delivery week <37 weeks), PROM (rupture of the membranes before delivery), stillbirth (the fetus has no signs of life after birth at 28 weeks gestational age and later), meconium-stained amniotic fluid (MSAF) (amniotic fluid is contaminated with meconium), and peripartum infection. Neonatal outcomes included low birth weight (birth weight <2,500 g), neonatal asphyxia (Apgar score ≤7 at 1 and 5 min after birth), neonatal infections (laboratory-diagnosed bacterial infection and/or the presence of clinical signs of infection) (Chan et al., 2015), and neonatal intensive care unit (NICU) admission.

Peripartum infection refers to the infection occurring intrapartum and postpartum (WHO Guidelines Approved by the Guidelines Review Committee, 2015). Patients with peripartum infection can have the infection of genital tract, urinary tract, breast, respiratory tract, surgical wound, bacteremia, and sepsis (Karsnitz, 2013; Song et al., 2020). Peripartum infection includes two or more of the following symptoms and signs of pelvic pain, fever, abnormal vaginal discharge, abnormal smell/foul odor discharge or delay in uterine involution (WHO Guidelines Approved by the Guidelines Review Committee, 2015). Peripartum infection was treated according to international guidelines (WHO Guidelines Approved by the Guidelines Review Committee, 2015).

### Statistical Methods

Statistical analysis was performed using SPSS version 24.0. Categorical variables were represented using the Chi-square test. Dichotomous logistic regression was used to identify risk factors for women with mixed vaginitis and peripartum infection. Statistical significance was set at *P* <0.05.

## Results

### Prevalence and Clinical Characteristics of Mixed Vaginitis

Among 1,674 women, 311 (18.58%) had vaginitis, namely, 245 (14.64%) single vaginitis and 66 (3.94%) mixed vaginitis. Among the women with mixed vaginitis, 1.49% (25/1,674) had AV + BV, 1.14% (19/1,674) had VVC + BV, 1.19% (20/1,674) had VVC + AV, 0.06% (1/1,674) had AV + trichomoniasis, and 0.06% (1/1,674) had VVC + AV + BV (**Table 1**). The clinical characteristics of the women with mixed vaginitis are summarized in **Table 2**.

**Table 1:** Prevalence of single and mixed vaginitis in pregnant women.

Diagnosis	No.	%
Nonvaginitis	1,363	81.42
Single VVC	137	8.18
Single BV	58	3.46
Single AV	47	2.81
Single Trichomoniasis	3	0.18
AV + BV	25	1.49
VVC + BV	19	1.14
VVC + AV	20	1.19
AV + Trichomoniasis	1	0.06
VVC + AV + BV	1	0.06
Total	1,674	100

**Table 2:** Clinical characteristics, vaginal microecological characteristics, symptoms and signs, and maternal–neonatal outcomes of women with mixed vaginitis during late pregnancy.

	Nonvaginitis n (%)	BV n (%)	AV n (%)	VVC n (%)	Trichomoniasis n (%)	BV + AV n (%)	BV + VVC n (%)	VVC + AV n (%)	AV + Trichomoniasis n (%)	VVC + AV + BV n (%)	Single Vaginitis n (%)	Mixed Vaginitis n (%)
**Sociodemographic Characteristics**												
Age, years ≥35	409 (30.0)	18 (31.0)	9 (19.1)	28 (20.4)	1 (33.3)	9 (36.0)	1 (5.3)^2^	6 (30.0)	0	0	56 (22.9)	16 (24.2)
Educational status < college	148 (10.9)	8 (13.8)	5 (10.6)	12 (8.8)	1 (33.3)	1 (4.0)	2 (10.5)	3 (15.0)	0	0	26 (10.6)	6 (9.1)
**Past Medical History**												
Positive of oral glucose tolerance test during pregnancy	170 (12.5)	6 (10.3)	9 (19.1)	22 (16.1)	1 (33.3)	5 (20.0)	6 (31.6)^2^	3 (15.0)	0	0	38 (15.5)	14 (21.2)*
History of vaginitis during pregnancy	130 (9.5)	11 (19.0)	8 (17.0)	30 (21.9)	1 (33.3)	8 (32.0)^1^	8 (42.1)^2^	9 (45.0)^3,f^	0	0	50 (20.4)	25 (37.9)^* ,#^
History of genital infections before pregnancy	348 (25.5)	13 (22.4)	10 (21.3)	44 (32.1)	1 (33.3)	4 (16.0)	5 (26.3)	11 (55.0)^3, e,f^	0	0	68 (27.8)	20 (30.3)
**Fertility History**												
Gravidity												
1	604 (44.3)	26 (44.8)	19 (40.4)	67 (48.9)	2 (66.7)	12 (48.0)	8 (42.1)	8 (40.0)	1 (100.0)	1 (100.0)	114 (46.5)	30 (45.5)
2–3	633 (46.4)	29 (50.0)	24 (51.1)	59 (43.1)	0	12 (48.0)	9 (47.4)	8 (40.0)	0	0	112 (45.7)	29 (43.9)
≥4	126 (9.2)	3 (5.2)	4 (8.5)	11 (8.0)	1 (33.3)	1 (4.0)	2 (10.5)	4 (20.0)	0	0	19 (7.8)	7 (10.6)
Parity												
0	916 (67.2)	44 (75.9)	31 (66.0)	99 (72.3)	2 (66.7)	19 (76.0)	12 (63.2)	13 (65.0)	1 (100.0)	1 (100.0)	176 (71.8)	46 (69.7)
1	418 (30.7)	14 (24.1)	13 (27.7)	35 (25.5)	1(33.3)	6 (24.0)	7 (36.8)	6 (30.0)	0	0	63 (25.7)	19 (28.8)
≥2	29 (2.1)	0	3 (6.4)	3 (2.2)	0	0	0	1 (5.0)	0	0	6 (2.4)	1 (1.5)
Abortion												
0	819 (60.1)	34 (58.6)	29 (61.7)	81 (59.1)	2 (66.7)	19 (76.0)	13 (68.4)	12 (60.0)	1 (100.0)	1 (100.0)	146 (59.6)	46 (69.7)^#^
1	345 (25.3)	19 (32.8)	12 (25.5)	43 (31.4)	0	3 (12.0)	2 (10.5)	4 (20.0)	0	0	74 (30.2)	9 (13.6)
≥2	199 (14.6)	5 (8.6)	6 (12.8)	13 (9.5)	1 (33.3)	3 (12.0)	4 (21.1)	4 (20.0)	0	0	25 (10.2)	11 (16.7)
**Personal Hygiene Habits**												
Panty liner use	234 (17.2)	8 (13.8)	8 (17.0)	30 (21.9)	1 (33.3)	4 (16.0)	5 (26.3)	5 (25.0)	1 (100.0)	1 (100.0)	47 (19.2)	16 (24.2)
External hemorrhoids	462 (33.9)	30 (51.7)	21 (44.7)	46 (33.6)	0	11 (44.0)	6 (31.6)	9 (45.0)	0	1 (100.0)	97 (39.6)	27 (40.9)
**Sexual Experience**												
Sex during pregnancy	465 (34.1)	23 (39.7)	9 (19.1)	53 (38.7)	2 (66.7)	9 (36.0)	12 (63.2)^2,d^	4 (20.0)	1 (100.0)	0	87 (35.5)	26 (39.4)
Number of sexual partners												
1	1,087 (79.8)	42 (72.4)	43 (91.5)	113 (82.5)	1 (33.3)	20 (80.0)	13 (68.4)	16 (80.0)	0	1 (100.0)	199 (81.2)	50 (75.8)
2–3	265 (19.4)	15 (25.9)	4 (8.5)	21 (15.3)	2 (66.7)	4 (16.0)	6 (31.6)	4 (20.0)	1 (100.0)	0	42 (17.1)	15 (22.7)
≥4	1 (0.8)	1 (1.7)	0	3 (2.2)	0	1 (4.0)	0	0	0	0	4 (1.6)	1 (1.5)
**Vaginal Microecological Characteristics**												
LBG												
I/IIa	1,320 (96.8)	0	0	120 (87.6)	3 (100.0)	0^1,b^	0^2,d^	0^3,e^	0^4^	0^5,g^	123 (50.2)	0*^, #^
IIb	43 (3.2)	5 (8.6)	31 (66.0)	17 (12.4)	0	0	0	15 (75.0)	0	0	53 (21.6)	15 (22.7)
III	0	53 (91.4)	16 (34.0)	0	0	25 (100.0)	19 (100.0)	5 (25.0)	1 (100.0)	1 (100.0)	69 (28.2)	51 (77.3)
Nugent score												
0–3	1,331 (97.7)	0	19 (40.4)	122 (89.1)	3 (100.0)	0^1,b^	0^2,d^	5 (25.0)^3,e^	0^4^	0^5, g,h^	144 (58.8)	5 (7.6)*^, #^
4–6	32 (2.3)	0	28 (59.6)	15 (10.9)	0	0	0	15 (75.0)	1 (100.0)	0	43 (17.6)	16 (24.2)
≥7	0	58 (100.0)	0	0	0	25 (100.0)	19 (100.0)	0	0	1 (100.0)	58 (23.7)	45 (68.2)
AV score												
0–2	1,363 (100.0)	58 (100.0)	0	137 (100.0)	3 (100.0)	0^1,a^	19 (100.0)	0^3,e^	0^4^	0^5, g,i^	198 (80.8)	19 (28.8)*^, #^
≥3	0	0	47 (100.0)	0	0	25 (100.0)	0	20 (100.0)	1 (100.0)	1 (100.0)	47 (19.2)	47 (71.2)
**Symptoms and signs**												
Increased discharge	604 (44.3)	30 (51.7)	25 (53.2)	83 (60.6)	1 (33.3)	14 (56.0)	14 (73.7)^2^	15 (75.0)^3^	1 (100.0)	0	139 (56.7)	44 (66.7)^*^
Genital itching	180 (13.2)	10 (17.2)	3 (6.4)	64 (46.7)	1 (33.3)	1 (4.0)	12 (63.2)^2,c^	15 (75.0)^3, e,f^	0	1 (100.0)	78 (31.8)	29 (43.9)^*^
Genital burning	34 (2.5)	0	0	9 (6.6)	1 (33.3)	0	2 (10.5)	4 (20.0)^3,f^	0	0	10 (4.1)	6 (9.1)^*^
Red and edematous vulva	58 (4.3)	3 (5.2)	6 (12.8)	27 (19.7)	1 (33.3)	0	7 (36.8)^2,c^	6 (30.0)^3^	1 (100.0)^4^	1 (100.0)^5^	37 (15.1)	15 (22.7)*
Vaginal erythema	130 (9.5)	11 (19.0)	16 (34.0)	75 (54.7)	1 (33.3)	10 (40.0)^1,a^	13 (68.4)^2,c^	17 (85.0)^3, e,f^	1 (100.0)	1 (100.0)	103 (42.0)	42 (63.6)*^, #^
Yellow discharge	396 (29.1)	20 (34.5)	32 (68.1)	91 (66.4)	3 (100.0)	18 (72.0)^1,a^	16 (84.2)^2,c^	19 (95.0)^3, e,f^	0	1 (100.0)	146 (59.6)	54 (81.8)*^, #^
Thick discharge	218 (16.0)	8 (13.8)	22 (46.8)	69 (50.4)	1 (33.3)	9 (36.0)^1,a^	10 (52.6)^2,c^	14 (70.0) ^3^	0	1 (100.0)	100 (40.8)	34 (51.5)*
Malodor	100 (7.3)	15 (25.9)	10 (21.3)	21 (15.3)	0	10 (40.0)^1^	7 (36.8)^d^	3 (15.0)	1 (100.0)	0	46 (18.8)	21 (31.8)*^, #^
**Maternal-Neonatal Outcomes**												
Delivered by cesarean section	827 (60.7)	37 (63.8)	22 (46.8)	81 (59.1)	2 (66.7)	12 (48.0)	9 (47.4)	13 (65.0)	0	1 (100.0)	142 (58.0)	35 (53.0)
PROM	260 (19.1)	11 (19.0)	11 (23.4)	14 (10.2)	1 (33.3)	3 (12.0)	1 (5.3)	5 (25.0)	0	0	37 (15.1)	9 (13.6)
Preterm	106 (7.8)	3 (5.2)	4 (8.5)	15 (10.9)	0	0	1 (5.3)	2 (10.0)	0	0	22 (9.0)	3 (4.5)
Stillbirth	2 (0.1)	0	0	0	0	0	0	0	0	0	0	0
Peripartum infection	19 (1.4)	1 (1.7)	1 (2.1)	1 (0.7)	0	1 (4.0)	0	3 (15.0)^3,e^	0	0	3 (1.2)	4 (6.1)*
Neonatal infection	185 (13.6)	5 (8.6)	7 (14.9)	27 (19.7)	0	4 (16.0)	4 (21.1)	3 (15.0)	0	0	39 (15.9)	11 (16.7)
Meconium-stained amniotic fluid	166 (12.2)	8 (13.8)	6 (12.8)	16 (11.7)	0	4 (16.0)	4 (21.1)	4 (20.0)	1 (100.0)	0	30 (12.2)	13 (19.7)
Low birth weight	83 (6.1)	3 (5.2)	3 (6.4)	11 (8.0)	0	1 (4.0)	1 (5.3)	3 (15.0)	0	0	17 (6.9)	5 (7.6)
Neonatal asphyxia	23 (1.7)	2 (3.4)	0	1 (0.7)	0	1 (4.0)	0	1 (5.0)	0	0	3 (1.2)	2 (3.0)
NICU	286 (21.0)	6 (10.3)	6 (12.8)	35 (25.5)	0	2 (8.0)	6 (31.6)	3 (15.0)	0	1 (100.0)	47 (19.2)	12 (18.2)

1: P <0.05, Nonvaginitis vs. BV + AV; 2: P <0.05, Nonvaginitis vs. VVC + BV; 3: P <0.05, Nonvaginitis vs. VVC + AV; 4: P <0.05, Nonvaginitis vs. Trichomoniasis + AV; 5: P <0.05, Nonvaginitis vs. VVC + AV + BV; a: P <0.05, BV vs. BV + AV; b: P <0.05, AV vs. BV + AV; c: P <0.05, BV vs. BV + VVC; d: P <0.05, VVC vs. BV + VVC; e: P <0.05, VVC vs. VVC + AV; f: P <0.05, AV vs. VVC + AV; g: P <0.05, VVC vs. VVC + AV + BV; h: P <0.05, AV vs. VVC + AV + BV; i: P <0.05, BV vs. VVC + AV + BV; *P <0.05, Nonvaginitis vs. Mixed vaginitis; ^#^P <0.05, Single vaginitis vs. Mixed vaginitis.

### Symptoms and Signs of Mixed Vaginitis

The symptoms and signs of mixed vaginitis are more complicated than those of single vaginitis (**Table 2**). The mixed vaginitis group had more obvious symptoms and signs, namely, increased discharge, genital itching, genital burning, red and edema vulva, vaginal erythema, yellow discharge, thick discharge, and malodor, than had the normal group. Furthermore, the occurrence of inflammation signs such as vaginal erythema (63.6% *vs.* 42.0%), yellow discharge (81.8% *vs*. 59.6%), and malodor (31.8% *vs*. 18.8%) in patients with mixed vaginitis were significantly different from those in patients with single vaginitis (*P* <0.05).

When we analyzed each form of mixed vaginitis, we found that vaginal erythema (40.0% *vs.* 19.0%), yellow discharge (72.0% *vs.* 34.5%), and thick discharge (36.0% *vs.* 13.8%) were more obvious in AV + BV than in BV (*P* <0.05). Genital itching (63.2% *vs.* 17.2%), red and edematous vulva (36.8% *vs.* 5.2%), vaginal erythema (68.4% *vs*. 19.0%), yellow discharge (84.2% *vs.* 34.5%), and thick discharge (52.6% *vs.* 13.8%) were more obvious in BV + VVC than in BV (*P* <0.05). We also found that malodor was more obvious in BV + VVC than in VVC (36.8% *vs.* 15.3%, *P* <0.05). For VVC + AV, we found that genital itching (75.0% *vs*. 46.7%), vaginal erythema (85.0% *vs.* 54.7%), and yellow discharge (95.0% *vs*. 66.4%) were more obvious in VVC + AV than in VVC (*P* <0.05). Furthermore, we found that genital itching (75.0% *vs.* 6.4%), genital burning (20.0% *vs.* 0%), vaginal erythema (85.0% *vs.* 34.0%), and yellow discharge (95.0% *vs.* 68.1%) were more obvious in VVC + AV than in AV (*P* <0.05).

### Risk Factors for Mixed Vaginitis

With women in the normal group as controls, a history of vaginitis during pregnancy (OR = 5.637, 95% CI: 3.314–9.580) was an independent risk factor for developing mixed vaginitis in late pregnancy (**Table 3**). When we analyzed each form of mixed vaginitis, we found that a history of vaginitis during pregnancy (OR = 4.463, 95% CI: 1.890–10.543) was an independent risk factor for developing BV + AV in late pregnancy. For BV + VVC, we found that a positive oral glucose tolerance test during pregnancy (OR = 3.573, 95% CI: 1.293–9.869), a history of vaginitis during pregnancy (OR =5.578, 95% CI: 2.165–14.369), and sex during pregnancy (OR = 2.887, 95% CI: 1.105–7.545) were an independent risk factors for developing BV + VVC in late pregnancy. For VVC + AV, we found that a history of vaginitis during pregnancy (OR = 6.300, 95% CI: 2.504–15.849) and a history of genital infections before pregnancy (OR = 2.668, 95% CI: 1.067–6.674) were an independent risk factors for developing VVC + AV in late pregnancy. We did not analyze risk factors of AV + trichomoniasis and VVC + AV + BV as we cannot use logistic regression analysis in the presence of only one case in both groups. Also, we did not find indicators that were statistically significant on the Chi-square test between AV + trichomoniasis group and VVC + AV + BV group and each form of single vaginitis in **Table 2**.

**Table 3:** Risk factors associated with mixed vaginitis during late pregnancy.

Risk factor	Mixed vaginitis *vs.* Nonvaginitis				BV + AV *vs.* Nonvaginitis				BV + VVC *vs.* Nonvaginitis				VVC + AV *vs.* Nonvaginitis			
	Univariate analysis		Multivariate analysis		Univariate analysis		Multivariate analysis		Univariate analysis		Multivariate analysis		Univariate analysis		Multivariate analysis	
	OR (95% CI)	*P*	OR (95% CI)	*P*	OR (95% CI)	*P*	OR (95% CI)	*P*	OR (95% CI)	*P*	OR (95% CI)	*P*	OR (95% CI)	*P*	OR (95% CI)	*P*
Age																
<35	1	0.391			1	0.519			1	0.047	1	0.073	1	0.999		
≥35	0.746 (0.420–1.326)				1.312 (0.575–2.993)				0.130 (0.017–0.974)		0.156 (0.020–1.189)		1.000 (0.381–2.620)			
Positive of oral glucose tolerance test during pregnancy																
No	1	0.041	1	0.092	1	0.267			1	0.019	1	0.014	1	0.735		
Yes	1.899 (1.025–3.483)		1.716 (0.951–3.218)		1.754 (0.650–4.736)				3.239 (1.215–8.635)		3.573 (1.293–9.869)		1.238 (0.359–4.270)			
History of vaginitis during pregnancy																
No	1	0.000	1	0.000	1	0.001			1	0.000	1	0.000	1	0.000	1	0.000
Yes	5.783 (3.407–9.817)		5.637 (3.314–9.580)		4.463 (1.890–10.543)				6.898 (2.726–17.456)		5.578 (2.165–14.369)		7.760 (3.157–19.073)		6.300 (2.504–15.849)	
Sex during pregnancy																
No	1	0.379			1	0.844			1	0.012	1	0.031	1	0.195		
Yes	1.255 (0.757–2.083)				1.086 (0.476–2.477)				3.311 (1.295–8.465)		2.887 (1.105–7.545)		0.483 (0.160–1.452)			
History of genital infections before pregnancy																
No	1	0.388			1	0.284			1	0.938			1	0.005	1	0.036
Yes	1.268 (0.740–2.174)				0.556 (0.189–1.630)				1.042 (0.372-2.913)				3.565 (1.465–8.675)		2.668 (1.067–6.674)	
Panty liner use																
No	1	0.142			1	0.878			1	0.301			1	0.362		
Yes	1.544 (0.864–2.758)				0.919 (0.313–2.702)				1.723 (0.615–4.830)				1.608 (0.579–4.468)			
External hemorrhoids																
No	1	0.242			1	0.294			1	0.832			1	0.302		
Yes	1.350 (0.816–2.233)				1.532 (0.690–3.402)				0.900 (0.340–2.383)				1.596 (0.657–3.878)			

### Microbiological Characteristics of Mixed Vaginitis

The vaginal microecological characteristics of women with mixed vaginitis are summarized in **Table 2**. The results show that under the microscope, the characteristics of the vaginal microbiota of patients with mixed vaginitis in the third trimester of pregnancy are complicated, and they differ from those of single vaginitis. Patients with mixed vaginitis showed decreased *Lactobacillus* and increased vaginitis indicators, such as mixed fungal and bacterial vaginitis and aerobic mixed with anaerobic bacterial infection. The same results were also found in BV + AV, BV + VVC, VVC + AV, and VVC + AV + BV groups when compared to each form of single vaginitis, such as BV, AV, and VVC.

Vaginal pathogens were isolated from 22 patients with mixed vaginitis (**Table 4**). Bacterial isolates from the vaginal discharge of patients with mixed bacterial vaginitis, were mainly pathogens of AV and BV, such as *G. vaginalis*, *S. anginosus*, and *S. epidermidis*. The pathogenic isolates from the vaginal discharge of patients in the mixed fungus and bacteria vaginitis group mainly included *C. albicans*, followed by *S. anginosus*, *E. faecalis*, *S. hemolyticus*, *S. aureus*, *S. agalactiae*, and *S. simulans*. The antimicrobial susceptibility test of isolated bacteria in patients with mixed vaginitis showed that the resistance rate to erythromycin was 83.3%, to tetracycline was 66.7%, to clindamycin was 33.3%, to levofloxacin was 16.7%, and to penicillin was 16.7%. We did not find any antimicrobial resistance to *C. albicans*.

**Table 4:** Microbial isolation of pathogens in mixed vaginitis during late pregnancy.

VVC + BV	VVC + AV	AV + BV
n = 7	n = 7	n = 8
*Candida albicans*, n = 3	*Candida albicans*, n = 5	*Gardnerella vaginalis*, n = 2
*Streptococcus anginosus*, n = 1	*Staphylococcus hemolyticus*, n = 1	*Streptococcus anginosus*, n = 1
*Staphylococcus aureus*, n = 1	*Enterococcus faecalis*, n = 1	*Staphylococcus epidermidis*, n = 1
*Streptococcus agalactiae*, n = 1	No colony, n = 1	No colony, n = 4
*Staphylococcus simulans*, n = 1		

### Adverse Pregnancy Outcomes of Women With Mixed Vaginitis

Peripartum infection was the main adverse outcome in women with mixed vaginitis. Compared with women in the normal group (6.1% *vs*. 1.4%), the incidence of peripartum infection in women in the mixed vaginitis group was higher (*P <*0.05) (**Table 2**). Compared with women in the single vaginitis group (6.1% *vs.* 1.2%), the incidence of peripartum infection in women with mixed vaginitis was also higher (*P* = 0.06). When we analyzed each form of mixed vaginitis, we found that women with VVC + AV had a higher rate of peripartum infection than women without vaginitis (15.0% *vs*. 1.4%) and women with VVC (15.0% *vs.* 0.7%) (*P <*0.05).

Mixed vaginitis increases the risk of peripartum infection. When women without peripartum infection were used as controls, mixed vaginitis in the third trimester (15.4% *vs*. 3.8%, *P* <0.05, OR=3.985, 95%CI:1.214-13.079), PROM (50.0% *vs*. 17.8%, *P* <0.05, OR=5.058, 95%CI:2.280-11.222), and meconium-stained amniotic fluid (42.3% *vs*. 12.0%, *P* <0.05, OR=5.387, 95%CI:2.375-12.218) were risk factors for peripartum infection (**Table 5**). We did not analyze the risk factor of each form of mixed vaginitis for the contribution of peripartum infection as some groups had a few cases.

**Table 5:** Risk factors associated with peripartum infection.

	Peripartum infection	Non-peripartum infection	Peripartum infection *vs.* Nonperipartum infection			
	(n = 26)	(n = 1648)	Univariate analysis		Multivariate analysis	
	No. (%)	No. (%)	OR (95%CI)	*P*	OR (95%CI)	*P*
Positive of oral glucose tolerance test during pregnancy						
No	20(76.9)	1432(86.9)	1	0.144		
Yes	6(23.1)	216(13.1)	1.989(0.790-5.008)			
Vaginitis						
Normal	19(73.1)	1344(81.6)	1		1	
Single vaginitis	3(11.5)	242(14.7)	0.877(0.258-2.986)	0.834	0.916(0.264-3.175)	0.890
Mixed vaginitis	4(15.4)	62(3.8)	4.564(1.507-13.818)	0.007	3.985(1.214-13.079)	0.023
External hemorrhoids						
No	16(61.5)	1072(65.0)	1	0.710		
Yes	10(38.5)	576(35.0)	1.163(0.524-2.580)			
Vaginal delivery						
No	20(76.9)	984(59.7)	1	0.083		
Yes	6(23.1)	664(40.3)	0.445 (0.178-1.113)			
PROM						
No	13(50.0)	1355(82.2)	1	0.000	1	0.000
Yes	13(50.0)	293(17.8)	4.625(2.122-10.079)		5.058(2.280-11.222)	
Meconium-stained amniotic fluid						
No	15(57.7)	1450(88.0)	1	0.000	1	0.000
Yes	11(42.3)	198(12.0)	5.370(2.432-11.858)		5.387(2.375-12.218)	

Pathogenic isolates of patients with peripartum infection are shown in **Table 6**. *E. coli* is the main pathogen that causes peripartum infection. All patients with a peripartum infection were treated successfully and discharged.

**Table 6:** Pathogenic isolation characteristics of patients with peripartum infection.

	Non-vaginitis (n = 19)	Single vaginitis (n=3)	Mixed vaginitis (n = 4)	Total isolates
**Blood culture**				
*E. coli*	6			6
*G. vaginalis*	1	1		2
No colony	7		4	
**Vaginal discharge culture**				
*E. coli*	1			1
*Lactobacillus delbruckii*	1			1
*S. hemolyticus* + *E. coli*	1			1
*E. faecalis*		1		1
Corynebacterium		1		1
*C. albicans*			2	2
*S. epidermidis*	1			1
No colony	5	1		
**Intrauterine discharge culture**				
*E. coli*	1			1
*S. hemolyticus*	1			1
**Wound secretion culture**				
*E. coli*	1			1
**Urine culture**				
No colony	1	1	1	
**Cervical discharge culture**				
No colony	2			
**Placenta swab culture**				
No colony	1			

## Discussion

Our study is a comprehensive analysis of the clinical features, microbiome characteristics, and pregnancy outcomes of mixed vaginitis during late pregnancy. Our research found that the history of vaginitis in the first and second trimesters of pregnancy increased the risk of mixed vaginitis in the third trimester. This indicates that mixed vaginitis is caused by long-term vaginal dysbiosis. Mixed vaginitis has complicated vaginal microbiota. Under the microscope, mixed vaginitis was indicated by a decrease in *Lactobacillus* and an increase in vaginitis scores. Bacterial isolation from vaginal discharge of patients in the mixed vaginitis group showed the presence of both aerobic and anaerobic bacteria in the mixed bacteria vaginitis group, and both fungi and bacteria were detected in the mixed fungus and bacteria vaginitis group. These results indicate that there is a complex interaction between microorganisms that promote the occurrence of mixed vaginitis. In the clinical manifestations of mixed vaginitis, the signs of vaginal inflammation (such as vaginal erythema, yellow discharge, and malodor) are more obvious than those of single vaginitis, especially in VVC + AV. Ultimately, mixed vaginitis in the third trimester, mainly in VVC + AV, led to an increased incidence of peripartum infection. Therefore, more attention should be paid to mixed vaginitis during pregnancy.

Immunity changes during pregnancy (Amir et al., 2020; Graham et al., 2021), and this change also increases the susceptibility for infectious diseases of pregnant women (Robinson and Klein, 2012). Existing studies have shown that the prevalence of mixed infections in non-pregnant women is lower than that in pregnant women. The prevalence rate of mixed infection was 6.5–61.0% during pregnancy (Shrestha et al., 2011; Abdelaziz et al., 2014; Han et al., 2019) and 2.4–10.0% for non-pregnant women (Karaer et al., 2005; Salinas et al., 2020; Hillier et al., 2021). Our study found that the prevalence rate of mixed vaginitis in late pregnancy was 3.9% (66/1,674). The prevalence rate of mixed vaginitis of our study was lower than that in pregnant women of existing studies. The reason for the different results may be associated with the different types of mixed vaginal infection involved in different researches. Our research found that the prevalence rate of AV + BV was 1.49%, while Han et al. found that the prevalence rate of AV + BV was 0.64% during pregnancy (Han et al., 2019). Our research found that the prevalence rate of BV + VVC was 1.14%, while other studies found that was 1.3–6.0% during pregnancy (Govender et al., 1996; Kiss et al., 2010; Shrestha et al., 2011; Han et al., 2019). Our research found that the prevalence rate of VVC + AV was 1.19%, while other studies found that was 3.53–4.49% during pregnancy (Mobasheri et al., 2014; Han et al., 2019). Our research found that the prevalence rate of VVC + AV + BV was 0.06%, while Han et al. found that was 1.60% during pregnancy (Han et al., 2019). We did not find that any research reported the prevalence rate of AV + trichomoniasis. However, other studies have reported the prevalence rate of VVC + trichomoniasis was 0.16-2.38% (Govender et al., 1996; Han et al., 2019), BV + VVC + trichomoniasis was 0.06-1.79%, and BV + trichomoniasis was 0.12-14.29% during pregnancy (Govender et al., 1996; Kiss et al., 2010). Those cases were not found in our research. Different researches reported different prevalence rates in the same form of mixed infection. Meanwhile, different researches reported different types of mixed infection. Both reasons may lead to the different prevalence rates between our study and other studies.

Studies have shown that a history of vaginitis is a risk factor for vaginitis in pregnant and non-pregnant women (Bradshaw et al., 2005; Amouri et al., 2011; Martin et al., 2013; Zeng et al., 2018; Han et al., 2019; Kim et al., 2020). Schwebke et al. found that more women with unstable vaginal flora have a history of BV than do women with stable vaginal flora (44% *vs.* 12%, *P* <0.05) (Schwebke et al., 1999). Our research found that vaginal infection in the first and second trimesters was the main cause of mixed vaginitis in the third trimester. Vaginal infection in the first and second trimesters also increases the risk of each form of mixed vaginitis, such as AV + BV, BV + VVC, and VVC + AV. The susceptibility for infectious diseases increased which was influenced by pregnancy (Robinson and Klein, 2012), women with a history of vaginitis are susceptible to vaginitis (Giraldo et al., 2000), and the proinflammatory state in the third trimester increases the risk of recurrence of vaginitis (Mor et al., 2017). All these may lead to the occurrence of mixed vaginitis during late pregnancy and lead to more complicated vaginal dysbiosis. The role of human genetic polymorphisms in vaginitis or in pathogens can further explain why patients with a history of vaginitis are susceptible to mixed vaginitis (Nedovic et al., 2014). However, Sarah et al. found that the occurrence of VVC is not related to vaginal infection that occurred 60 days before the occurrence of VVC (Brown et al., 2019). The reason for the inconsistent results between previous studies and our study may be because this study only investigated the epidemiological characteristics of patients with asymptomatic VVC. However, the symptoms of vaginitis are indispensable in the diagnosis of vaginitis. In addition, Mengistie et al. found that there is no difference in the history of previous reproductive tract infections between patients with BV and those without BV during pregnancy (Mengistie et al., 2014). The reasons for the inconsistent results may be because this study investigated BV in all trimesters of pregnancy. However, the prevalence of vaginitis is different among the stages of pregnancy due to the different immune statuses in early, middle, and late pregnancy (Mor et al., 2017).

Women co-infected with complicated vaginal microbiota. Ana et al. found that women with vaginal coinfections had higher percentages of *Atopobium vaginae* and *Gardnerella* spp., and followed by *Mobiluncus mulieris*, *E. coli*, and *C. albicans* (Salinas et al., 2020). Brotman et al. found that women with trichomoniasis accompanied Nugent ≥7 had higher abundance of *Lactobacillus iners*, *Prevotella*, *Megasphaera*, *Sneathia*, *Atopobium*, *Diallister*, and *Lachnospira* (Brotman et al., 2012). Our study found that isolation of pathogens of the vaginal flora in patients with mixed vaginitis showed complex results, which are similar to that showed by microscopy diagnosis. Bacterial isolates of vaginal discharge of patients with AV + BV were mainly related to AV and BV bacteria, such as *G. vaginalis*, *S. anginosus*, and *S. epidermidis*. The pathogen isolates of the vaginal discharge of patients in the VVC + AV and VVC + BV group mainly included *C. albicans*, followed by *S. anginosus*, *E. faecalis*, *S. hemolyticus*, *S. aureus*, *S. agalactiae*, and *S. simulans*. The interactions between microorganisms play an important role in the inflammatory phenotype. Regarding mixed infection of *C. albicans* and aerobic bacteria, studies have found that a model of peritonitis in mice coinfected with *S. aureus* and *C. albicans* caused more severe infections, produced more macrophage inflammatory protein-1α, recruited more neutrophils, caused inflammation in the local abdominal cavity, and increased interleukin (IL)-6 and PGE2 levels in the local abdominal cavity than when the mice were infected with one bacteria (Peters and Noverr, 2013). In severe cases, these responses caused the death of the mouse (Nash et al., 2016). Regarding mixed infection of anaerobic and aerobic bacteria, studies have found that a model of urinary tract infections in mouse where exposure of the bladder to *G. vaginalis* leads to the activation of *E. coli* from latent bladder reservoirs, which further causes bladder epithelial apoptosis and exfoliation and IL-1 receptor-mediated kidney damage, leading to serious urinary tract infections (Gilbert et al., 2017). *In vivo* studies in another mixed infection model of aerobic and anaerobic bacteria have found that compared with MRSA-infected mice that are not infected with *Prevotella intermedia* supernatant, mice infected with hospital-acquired methicillin-resistant *S. aureus* (HA-MRSA) and *P. intermedia* supernatant had a significantly lower survival rate, a significantly higher lung bacterial load, and significantly higher expression of α-hemolysin in the lungs (Yamashita et al., 2020). A study on the inflammatory factors in vaginal secretions of patients with mixed infection of BV + trichomoniasis found that the inflammatory factors, regulated on activation, normal T cell expressed and secreted (RANTES), macrophage inflammatory protein (MIP)-3α, MIP-1β, monocyte chemotactic protein, and galectin-1, were elevated, indicating an obvious inflammatory phenotype in mixed infections (Fichorova et al., 2021).

The interaction between microorganisms plays an important role in leading to the phenotype of vaginal inflammation. Our study found that signs of vaginal erythema (63.6% *vs.* 42.0%), yellow discharge (81.8% *vs*. 59.6%), and malodor (31.8% *vs*. 18.8%) (*P <*0.05) in patients with mixed vaginitis were significantly different from those in patients with single vaginitis. Patients with mixed vaginal infection of AV + BV had more obvious signs of vaginal infection than patients with BV, while these signs were difficult to distinguish from patients with AV. Patients with mixed vaginal infection of VVC + BV had more obvious symptoms and signs of vaginal infection than patients with BV, while these signs were difficult to distinguish from patients with VVC. Patients with double infection of inflammatory vaginitis of VVC and AV had more severe inflammatory phenotype than patients with VVC and AV respectively. This implies that the symptoms and signs of inflammation in the vagina of patients with mixed vaginitis are more obvious than those in patients with single vaginitis, highlighting the more serious phenotype observed by the interaction between pathogens and also the interaction between pathogens and the human immune system. Similarly, in 2012, Fan et al. (2013) found that it was difficult to use symptoms to distinguish AV + BV infections from an AV infection, while they could be distinguished from BV. In 2011, Charles et al. (Rivers et al., 2011) found that vulvar pruritus, erythema, and thick curdy discharge in patients with BV + VVC infection were more obvious than those observed in patients with BV, but there was a slight difference when the findings were compared with that of patients with VVC. In 2021, Ellen et al. (van den Munckhof et al., 2021) found that malodor and vulval/vaginal erythema and edema had higher sensitivity point to BV + VVC, while in BV + VVC only vulval/vaginal erythema and edema can be obviously differentiated from BV. In 2011, Gondo et al. (2011) found that the symptoms and signs of vaginal infection in pregnant women with mixed vaginitis were more obvious than those in patients with single vaginitis, but these symptoms and signs could not be distinguished from those of patients with VVC. This may be related to the difficulty in distinguishing the related inflammatory symptoms of VVC infection from that of mixed vaginitis, as patients with VVC also shows symptoms such as vaginal erythema, yellow discharge, and pruritus. Fan et al. also highlights that inflammatory vaginitis exists in mixed infections, which is indistinguishable from the corresponding inflammatory vaginitis in single infections, such as mixed vaginitis of VVC versus single vaginitis of VVC and mixed vaginitis of AV versus single vaginitis of AV. However, BV is a non-inflammatory vaginosis, so VVC or AV mixed with a BV infection can be clearly distinguished from single BV. The above studies generally illustrate the severe and complex inflammatory phenotypes of patients with mixed vaginitis. This severe phenotype may be related to the enhanced virulence caused by the interaction between microorganisms, such as the interaction between *S. aureus* and *C. albicans* (Peters et al., 2012; Todd et al., 2019) and between *P. intermedia* and MRSA strain (Yamashita et al., 2020). However, some studies have found that the interaction between microorganisms does not cause an inflammatory phenotype (Chaisilwattana and Monif, 1995; Nair et al., 2001; Thein et al., 2007; Bandara et al., 2013; Brosnahan et al., 2013). The reason for the inconsistent results of these studies is not yet clear. Whether the interaction between microorganisms causes disease in the host requires further research.

Postpartum infections impose a huge burden on socioeconomic and maternal–neonatal health (Boushra and Rahman, 2022). Approximately 5–7% of postpartum women will develop postpartum infections. Abnormal vaginal flora during pregnancy is associated with puerperal infections (Zhang et al., 2018; Han et al., 2019). In 2016, Subramaniam et al. (2016) studied the characteristics of the vaginal flora of patients with puerperal infection in the second trimester and found that the vaginal flora of patients with puerperal infection was complicated and that the number of some of the species, such as BVAB1, *Pleomorphomonas*, *Acinetobacter*, and *Agrobacterium*, had significantly increased. Similar to our study, mixed infection was associated with an increased risk of peripartum infection, which may be related to ascending pathogens from the lower genital tract. In our study, the types of vaginitis in patients with mixed vaginitis who developed puerperal infections were mainly VVC + AV. An *in vivo* experiment demonstrated that the fungal burden and the level of IL-1β, IL-6, and TNF-α were increased when *S. agalactiae* co-infected with pathogen of VVC in a mouse model (Yu et al., 2018). Wei et al. (Dai et al., 2019) conducted research on 380 women in late pregnancy and found that the puerperium infection was related to the colonization of GBS (AV pathogen) during pregnancy. The presence of pathogen of VVC and GBS simultaneously may increase the risk of puerperium infection.

Studies have shown that PROM increases the risk of puerperal infection. In 2020, Song et al. (2020) studied the risk factors for puerperal infection and found that PROM increased the risk of puerperal infection (OR: 2.44, 95% CI: 1.38–4.31). In 2019, Anna et al. (DeNoble et al., 2019) studied the influencing factors of postpartum infection and found that the incidence of PROM was higher in patients with postpartum infection than in patients without postpartum infection (37.5% *vs.* 18.1%, *P* = 0.03). In 2019, Demisse et al. (2019) studied the risk factors for puerperal sepsis and found that the main determinant of puerperal sepsis was PROM and prolonged PROM time (AOR: 3.73; 95% CI: 1.37–10.2). When the fetal membranes rupture during pregnancy, the integrity of the uterine cavity cannot be maintained, which may further lead to an ascending infection of the uterine cavity caused by microorganisms in the lower genital tract. In addition, the ascending infection of mixed vaginitis with a complicated vaginal microenvironment may further increase the burden of uterine cavity infection and lead to the occurrence of peripartum infection. The occurrence of meconium-stained amniotic fluid in pregnant women with intact fetal membranes is a risk factor for microbial invasion of the amniotic cavity (Romero et al., 2014). In 2003, Tran et al. (2003) found that meconium-stained amniotic fluid was associated with puerperal infection and that puerperal infection increased with the severity of meconium-stained amniotic fluid. Therefore, in the case of mixed vaginitis pathogens with ascending infection accompanied by meconium-stained amniotic fluid or PROM, the risk of puerperal infection is further increased. However, studies have found that the incidence of puerperal infection in patients with meconium-stained amniotic fluid is not significantly higher than that in patients with clear amniotic fluid, and PROM in patients with meconium-stained amniotic fluid is not higher than that in patients with clear amniotic fluid (Mazor et al., 1998). The inconsistency with our study results may be because this study did not investigate the role of vaginitis in adverse pregnancy outcomes.

*E. coli* was the main pathogen of postpartum infection in our study, which is similar to the results of other studies (O’Higgins et al., 2014; Knowles et al., 2015). Studies have found that reproductive tract infection is the main cause of perinatal sepsis, and gram-negative *E. coli* is the main pathogen of the infection (Duan et al., 2019). Song et al. analyzed the risk factors and pathogens that cause puerperal infection and found that similar to our study, PROM was one of the risk factors of puerperal infection (Song et al., 2020). Meanwhile the authors also found that *E. coli* was the primary pathogen, followed by *Enterococcus*, *G. vaginalis*, *S. aureus*, *Streptococcus*, *S. epidermidis*, and *C. albicans*.

This study has some limitations. In our study, pathogens were isolated from the vaginal discharge of patients with mixed vaginitis. The main findings were similar to those of the microbiota characteristics of the disease. However, the detection rate of BV-related bacteria in some patients with VVC + BV infection is low. The growth conditions of BV-related bacteria may be harsh and ordinary culture cannot be accurately identified, so culture is not the first choice for the diagnosis of BV (Gergova et al., 2013; van den Munckhof et al., 2019; Workowski et al., 2021) even though it is important to identify pathogens and to test the drug susceptibility of pathogen for further treatment of the diseases (Mulu et al., 2015; Tang et al., 2020). In addition, the treatment protocol of mixed vaginitis is not consensus, which may also be one of the reasons for peripartum infection in patients with mixed vaginitis. In the future, we will explore the treatment of mixed vaginitis and clarify the best treatment plan for the different types of mixed vaginitis. During pregnancy, the use of most drugs, such as quinolones, is limited (Stein et al., 2003). The development of therapeutic drugs suitable for the treatment of mixed vaginitis during pregnancy should be further clarified. In the future, we will sequence the vaginal discharge of patients with mixed vaginitis to study the microbial mechanisms of the interactions between different species. We will also develop an animal model of mixed vaginitis to determine its effect on pregnancy outcomes.

## Conclusion

Mixed vaginitis is adverse for pregnant women in late pregnancy. The prevalence rate of mixed vaginitis is 3.94%. A history of vaginitis during pregnancy was an independent risk factor for developing AV + BV, BV + VVC, and VVC + AV mixed infection in late pregnancy. Under the microscope, patients with mixed vaginitis showed decreased *Lactobacillus* and increased vaginitis indicators, such as BV + AV, BV + VVC, VVC + AV, VVC + AV + BV mixed infection when compared to each form of single vaginitis. Microbial isolates from the vaginal discharge of patients with mixed infection were also complicated meaning that there were complexed interactions between the microorganisms. These interactions may lead to severe inflammatory symptoms and signs. The mixed vaginitis group had more obvious inflammation signs, especially in AV + BV, VVC + BV, and VVC + AV, than the single vaginitis group. Peripartum infection was the main adverse outcome in women with mixed vaginitis, especially in VVC + AV. Pathogenic isolates of patients with peripartum infection are showed that *E. coli* is the main pathogen that causes puerperal infection. For the above results, we should pay more attention to mixed infection. Research on the pathogenic mechanism of and clinical intervention for mixed vaginitis is worthy of future exploration.

## Data Availability Statement

The raw data supporting the conclusions of this article will be made available by the authors, without undue reservation.

## Ethics Statement

The studies involving human participants were reviewed and approved by the Ethics Committee of Tianjin Medical University General Hospital. The patients/participants provided their informed consent to participate in this study.

## Author Contributions

CH, and FX conceived the study question, and all authors were involved in the study design. YY was involved in the statistical analysis while HRL, MD, and WX interpreted the results. HRL created the first draft of the manuscript. HRL, WQ, FT, HYL, CW, CH, and FX made substantial contributions to drafting the article and revising it critically. All authors listed have made a substantial, direct, and intellectual contribution to the work and approved it for publication.

## Funding

This work was supported by the Tianjin Municipal Science and Technology Commission Special Foundation for Science and Technology Major Projects in Control and Prevention of Major Diseases (Grant No. 18ZXDBSY00200), the General Project of the National Natural Science Foundation of China (Grant No. 82071674), the National Natural Science Foundation of China (Grant No. 82101705), the Natural Science Foundation of Tianjin Municipal Science and Technology (Grant No. 20JCYBJC00440), the Tianjin Health Science and Technology Project (Grant No. KJ20176; KJ20003), the Scientific Research Project of Tianjin Education Commission (Grant No. 2020KJ158), and Tianjin Key Medical Discipline (Specialty) Construction Project.

## Conflict of Interest

The authors declare that the research was conducted in the absence of any commercial or financial relationships that could be construed as a potential conflict of interest.

## Publisher’s Note

All claims expressed in this article are solely those of the authors and do not necessarily represent those of their affiliated organizations, or those of the publisher, the editors and the reviewers. Any product that may be evaluated in this article, or claim that may be made by its manufacturer, is not guaranteed or endorsed by the publisher.
